# Stent or Surgery for Malignant Low Bileduct Obstruction?

**DOI:** 10.1155/1997/21935

**Published:** 1997

**Authors:** Ingemar Ihse, Lars Hansson, Lars-Erik Hammarström, Eva Lindström

**Affiliations:** Department of Surgery University Hospital Lund S-221 85 Sweden

## Abstract

The development of non-surgical techniques for the relief of malignant low bileduct obstruction has cast doubt on the best way of relieving jaundice, particularly in patients fit for surgery whose life expectancy is more than a few weeks.

We did a randomised prospective controlled trial comparing endoscopic stent insertion and surgical biliary bypass in patients with malignant low bileduct obstruction. 204 patients were randomised (surgery 103, stent 101); 3 subsequently proved to have benign disease and were excluded, leaving 101 surgical and 100 stented patients for assessment. Technical success was achieved in 94 surgical and 95 stent patients, with functional biliary decompression obtained in 92 patients in both groups. In stented patients, there was a lower procedure-related mortality (3% *vs* 14%, *p*=0.01), major complication rate (11% *vs* 29%, *p*=0.02), and median total hospital stay (20 *vs* 26 days, *p*=0.001). Recurrent jaundice occurred in 36 stented patients and 2 surgical patients. Late gastric outlet obstruction occurred in 17% of stented patients and 7% of the surgical group. Despite the early benefits of stenting there was no significant difference in overall survival between the two groups (median survival: surgical 26 weeks; stented 21 weeks; *p*=0.065).

Endoscopic stenting and surgery are effective palliative treatments with the former having fewer early treatment-related complications and the latter fewer late complications.


2. Fan, S. T., Lo, C. M., Lai, E., Chu, K. M., Liu, C. L. and Wong, J. (1994). Perioperative nutritional support in patients undergoing hepatechtomy for hepatocellular carcinoma, *NEJM*, **331**, 1547–1552.
3. Veterans' Affairs Total Parenteral Nutrition Cooperative Study Group (1991). Perioperative total parenteral nutrition in surgical patients, *NEJM*, **325**, 525–532.
4. Sandström, R., Drott, C. and Hyltander, A., *et al.* (1993). the effect of postoperative intravenous feeding (TPN) on outcome following major surgery evaluated in a randomized study, *Annals of Surgery*, **217**, 185–195.
5. Brenan, M. F., Pisters, P. W. T. and Posner, M., *et al.* (1994). A prospective randomized study of total parenteral nutrition after major pancreatic resection for malignancy, *Annals of Surgery*, **222**, 436–444.
6. Fan, S.T., Lai, E. C. and Lo, C. M., *et al.* (1995). Hospital mortality of major hepatectomy for hepatocellular carcinoma associated with cirrhosis, *Archives of Surgery*, **130**, 198–203.
7. Lai, E. C., Fan, S. T. and Lo, C. M., *et al.* (1995). Hepatic resection for hepatocellular carcinoma, an audit of 343 patients, *Annals of Surgery*, **221**, 291–298.
8. Delany, H. M., John, J. and Teh, E. L., *et al.* (1994). Contrasting effects of identical nutrients given parenterally or enterally after 70% hepatectomy, *American Journal of Surgery*, **167**, 135–143.

Albert Bothe Jr. and Glenn Steele Jr.
Department of Surgery
Deaconess Hospital
Boston, MA 02215
United States of America

### STENT OR SURGERY FOR MALIGNANT LOW BILEDUCT OBSTRUCTION?

### ABSTRACT

*Smith, A.C., Dowsett, J.F., Russell, R.C.G., Hatfield, A.R.W. and Cotton, P.B. (1994) Randomised trial of endoscopic stenting versus surgical bypass in malignant low bileduct obstruction. The Lancet; **344**: 1655–1660.*

**The development of non-surgical techniques for the relief of malignant low bileduct obstruction has cast doubt on the best way of relieving jaundice, particularly in patients fit for surgery whose life expectancy is more than a few weeks.**

**We did a randomised prospective controlled trial comparing endoscopic stent insertion and surgical biliary bypass in patients with malignant low bileduct obstruction. 204 patients were randomised (surgery 103, stent 101); 3 subsequently proved to have benign disease and were excluded, leaving 101 surgical and 100 stented patients for assessment. Technical success was achieved in 94 surgical and 95 stent patients, with functional biliary decompression obtained in 92 patients in both groups. In stented patients, there was a lower procedure-related mortality (3% *vs* 14%, $p$=0.01), major complication rate (11% *vs* 29%, $p$=0.02), and median total hospital stay (20 *vs* 26 days, $p$=0.001). Recurrent jaundice occurred in 36 stented patients and 2 surgical patients. Late gastric outlet obstruction occurred in 17% of stented patients and 7% of the surgical group. Despite the early benefits of stenting there was no significant difference in overall survival between the two groups (median survival: surgical 26 weeks; stented 21 weeks; $p$=0.065).**

**Endoscopic stenting and surgery are effective palliative treatments with the former having fewer early treatment-related complications and the latter fewer late complications.**

**Lancet 1994; 344: 1655–60**

KEYWORDS: Endoprothesis, Biliary enteric bypass, bileduct, carcinoma pancreas

### PAPER DISCUSSION

Endoscopic treatment of malignant bile duct obstruction has become an accepted alternative to surgical bilioenteric by-pass during the last decade. There were, however, only two prospective randomized comparisons available[1,2] until Smith and associates published their study in 1994. They aimed at providing a basis for the patients to make fully informed decisions on whether their jaundice should be relieved endoscopically or surgically.

Unlike the previous two randomized studies[1,2] Smith and associates were able to collect a sufficient number of patients to make the results statistically reliable. Only patients in whom the tumor was deemed unresectable were included in the study and, thus, the question of the role of preoperative biliary drainage was not addressed.

A prerequisite for the use of non-surgical techniques for palliative purposes is that available imaging tests correctly distinguish between resectable and unresectable disease. We feel that Smith and associates, like several other authors, overemphasize the power and accuracy of such tests in this respect. We have to remember that data are generally published from expert centers and that even here the sensitivity obtained for different tests is far below 100% (3).

Therefore, patients who are otherwise fit will and should undergo a laparotomy by an experienced surgeon irrespective of whether the imaging tests suspect a locally unresectable lesion or not. The difficulties in even verifying the diagnosis preoperatively are demonstrated by Smith and associates who obtained histological or cytological proof in only two thirds of the patients.

In our department percutaneous cytodiagnosis was widely used during the 1970's and the first half of the 1980's, but due to the high proportion of false negative findings it is today only practised in those with unquestionably advanced disease to strengthen the basis for the discussion with the patient and his/her family on prognosis and palliation (4). In patients with metastatic disease preoperative staging is often accurate especially if cytology is done but the problematic clinical situation arises in the patient with suspected local unresectability who is otherwise healthy and fit for a major operation. Then an exploratory laparotomy should always be done preferably by a surgeon who is prepared to go on with a pancreaticoduodenectomy when appropriate.

The authors found an equal procedural and therapeutic success rate after endoscopic and surgical bypass, respectively. The procedure related mortality was significantly lower after endoscopic than after surgical bypass. However, the 30-day mortality was similar in the two groups. Thus, the patients in the worst general condition succumb early after surgery but after endoscopic treatment they die anyhow within one month, thus questioning the advantages of endoscopy in this particular respect. Furthermore, the postoperative mortality rate was higher than reported in some recent studies on palliative bilioenteric anastomoses[5,6,7]. One tentative reason for this could be that several of the surgical bypasses were done in different referring hospitals outside the Middlesex, whereas the endoscopy was fully in the hands of the experts of this hospital. There were fewer major early complications after endoscopy than after surgery whereas late complications, such as recurrent jaundice and gastric outlet obstruction, were more frequent in the endoscopy group. Surprisingly the rate of cholangitis attacks after the two procedures, respectively, were not given. There was no difference in overall survival between the two treatments.

The study by Smith and associates is important as it is prospective, randomized and of sufficient statistical power. However, as pointed out above, the unexpectedly high early mortality rate after biliodigestive bypass does not do full justice to surgery, even if the statistics are correct. Thefindings of the study, though, lend support to the strategy which is currently practised at many centers such as ours. All patients with a suspicion of extrahepatic biliary obstruction undergoERCP. If a malignant type of obstruction is found, stenting is done at the same session. Patients who turn out to suffer from definitely incurable disease will get no further treatment whereas those without signs of metastases or clearcut unresectability on imaging tests will have a laparotomy with the aim of resection. Staging laparoscopy is presently being evaluated but its clinical value and cost-effectiveness needs confirmation (8).

Surgery has the advantage of offering at the same time a definite evaluation of resectability, including frozen section biopsy of the tumor and of secondaries, resection or palliative procedures such as bilioenteric shunt, gastroenteroanastomosis or coelic bloc, and intraoperative radiotherapy (IORT) and/or clipping of the tumor outline if a locally unresectable lesion is planned for postoperative radiotherapy. Endoscopy currently only offers routine relief of jaundice. However, recently biliary-type metallic stents have been applied to relieve duodenal obstruction and the development of larger diameter devices has further improved this early encouraging experience (9). Also, there is some initial information on pain treatment by placement of palstic stents in pancreatic cancer patients with main duct obstruction. In carefully selected patients, the results are promising (10). For palliative purposes the endoscopic approach in a broader sense has also been applied laparoscopically as well as thoracoscopically.

Cholecystojejunostomy and gastroenteroanastomosis can be properly done via the laparoscopic route (11) and pain relief can be achieved by thoracoscopic splanchnicectomy (12). Thus, both surgery and endoscopy comprise a great armamentarium and possibilities which today and in the future may offer good and improved palliation to patients with malignant disease affecting the common bile duct and its surroundings.

Since the first endoscopic biliary drainages were done, the stent diameter has been successively increased in order to counteract the problem of clogging causing cholangitis and/or recurrent jaundice. Although Smith and associates used 10 FG plastic stents, the risk of recurrent biliary obstruction was significant. The maximum diameter of plastic stents is limited by the instrument channel and cannot be increased further than the 10 to 11.5 FG which is now standard. The self-expandable metallic stents may expand to 30 FG when in place in the bile duct. Randomized studies report prolonged patency although tumor ingrowth has now appeared as a cause of stent occlusion[13,14,15]. This problem will hopefully be overcome by silicone covering or with metallic stents which do not have an open framework.

There is fast development in both the surgical, especially the laparoscopic, and the endoscopic approachs to the palliative relief of malignant bile duct obstruction. The two techniques complement each other and the study by Smith and associates helps in defining their current roles.

### REFERENCES

1. Shepherd, A, H., Royle, G., Ross, A. P. R., Diba, A., Arthur, M. and Colin-Jones, D. (1988). Endoscopic biliary endoprosthesis in the palliation of malignant obstruction of the distal common bile duct: a randomized trial, *Br. J. Surg.*, **75**, 1166–1168.
2. Andersen, J. R., Sorensen, S. M., Kruse, A., Rallaer, M. and Matzen, P. (1989). Randomized trial of endoscopic endoprosthesis versus operative by-pass in malignant obstructive jaundice, *Gut*, **30**, 1132–1135.
3. Warshaw, A. L. and Swanson, R. S. (1988). Pancreatic cancer in 1988. Possibilities and probabilities, *Ann Surg*, **208**, 541–553.
4. Ihse, I. and Andrén-Sandberg, Å. (1993). Percutaneous fine-needle aspiration cytology; in Trede M, Carter DC (eds): Surgery of the pancreas. Edinburgh, Churchill Livingstone, pp. 121–125.
5. Potts, J. R III., Brougham, T. A. and Herman, R. E. (1990). Palliative operations for pancreatic cancer, *Am. J. Surg.*, **159**, 72–78.
6. de Rooij, P. D., Rogatko, A. and Brennan, M. (1991). Evaluation of palliative surgical procedures in unresectable pancreatic cancer, *Br J Surg*, **78**, 1053–1058.
7. Lillemoe, K. D., Sauter, P. K., Pitt, H. A., Yeo, C. J. and Cameron, J. L. (1993). Current status of surgical palliation of periampullary carcinoma, *Surg Gynaecol Obstet*, **176**, 1–10.
8. Andersson, R., Axelson, J., Ihse, I. and Andrén-Sandberg, A. (1995). Staging laparoscopy in pancreatic cancer, *Int J Pancreatol*, in press (abstract).
9. Carr-Locke, D. L. (1995). Pancreatic cancer: stenting versus surgery, *Dig Surg*, **11**, 366–371.
10. Costamagna, G., Gabbrielle, A., Mutignani, M., Perri, V. and Crucitti, F. (1993). Treatment of obstructive pain by endoscopic drainage in patients with pancreatic head carcinoma, *Endoscopy*, **39**, 770–773.
11. Shimi, S., Banting, S. and Cuschieri, A. (1992). Laparoscopy in the management of pancreatic cancer: endoscopic cholecystojejunostomy for advanced disease, *Br J Surg*, **79**, 317–319.
12. Andrén-Sandberg, Å., Zoucas, E., Gyllstedt, E., Lillo-Gill, R. and Ihse, I. (1994). Thoracoscopic splanchnicectomy in pancreatic pain, *Digestion*, **55**, 278 (abstract).
13. Knyrin, K., Wagner, H. J., Starck, E., Hertberg, A., Pausch, J. and Vakil, N. (1992). Metal oder Kunststoffendoprothesen bei malignem Verschlussikterus. Ein randomisierter und prospektive Vergleich, *Dtsch Med Wochenschr*, **117**, 847–853.
14. Carr-Locke, D. L., Ball, T. J., Connors, P. J., Cotton, P. B., Geenen, J. E., Hawes, R. H., Jowell, P. S., Kozarek, R. A., Lehman, G. A., Meier, P. B., Ostro, J. W., Shapiro, H. A., Silvis S. E. and Vennes, J. A. (1993). Multicenter randomized trial of Wallstent biliary endoprosthesis versus plastic stents, *Gastrointest Endosc*, **39**, 310.
15. Davids, P. H. P., Groen, A. K., Rauws, E. A. J., Tytgat, G. N. J. and Huibregtse, K. (1992). Randomized trial of self-expanding metal stents versus polyethylene stents for distal malignant biliary obstruction, *Lancet*, **340**, 1488–1492.

Ingemar Ihse, MD, PhD, Professor and Chairman
Lars Hansson, MD, PhD
Lars-Erik Hammarström, MD, PhD
Eva Lindström, MD, PhD

Department of Surgery
University Hospital
S-221 85 Lund
Sweden

